# Gamma Probe Guided Minimally Invasive Parathyroidectomy without Quick Parathyroid Hormone Measurement in the Cases of Solitary Parathyroid Adenomas

**DOI:** 10.4274/Mirt.69885

**Published:** 2013-04-05

**Authors:** Savaş Karyağar, Sevda S Karyağar, Orhan Yalçın, Enis Yüney, Mehmet Mülazımoğlu, Tevfik Özpaçacı, Oğuzhan Karatepe, Yaşar Özdenkaya

**Affiliations:** 1 Trabzon Kanuni Training and Research Hospital, Department of Nuclear Medicine, Trabzon, Turkey; 2 Okmeydanı Training and Research Hospital, Department of General Surgery, İstanbul, Turkey; 3 Okmeydani Training and Research Hospital, Department of Nuclear Medicine, Istanbul, Turkey

**Keywords:** Solitary parathyroid Adenoma, gamma probe, parathyroidectomy

## Abstract

**Objective:** In this study, our aim was to study the efficiency of gamma probe guided minimally invasive parathyroidectomy (GP-MIP), conducted without the intra-operative quick parathyroid hormone (QPTH) measurement in the cases of solitary parathyroid adenomas (SPA) detected with USG and dual phase 99mTc-MIBI parathyroid scintigraphy (PS) in the preoperative period.

**Material and Methods:** This clinical study was performed in 31 SPA patients (27 female, 4 male; mean age 51±11years) between February 2006 and January 2009. All patients were operated within 30 days after the detection of the SPA with dual phase 99mTc-MIBI PS and USG. The GP-MIP was done 90-120 min after the iv injection of 740 MBq 99mTc-MIBI. In all cases, except 1 patient, the GP-MIP was performed under local anesthesia; due to the enormity of size of SPA, then general anesthesia is chosen.

**Results:** The operation time was 30-60 min, mean 38,2±7 min. In the first postoperative day, there was a more than 50% decrease in PTH levels in all patients and all but one had normal serum calcium levels. Transient hypocalcemia was detected in one patient.

**Conclusion:** GP-MIP without intra-operative QPTH measurement is a suitable method in the surgical treatment of SPA detected by dual phase 99mTc-MIBI PS and USG.

**Conflict of interest:**None declared.

## INTRODUCTION

Patients with solitary parathyroid adenoma (SPA) detected by parathyroid scintigraphy (PS), having no thyroid nodule in the ultrasonography (USG), and without a history of familial hyperparathyroidism (HPT) or multiple endocrine neoplasia syndrome, constitute 60-70% of primer hyperparathyroidism (PHPT) cases. These cases are suitable for gamma probe guided minimally invasive parathyroidectomy (GP-MIP) (1). In recent years, due to highly sensitive and accurate detection of SPAs via preoperative PS and USG and the usage of intra-operative gamma probe and the measurement of intra-operative quick parathyroid hormone (QPTH), MIP is chosen by endocrine surgeons instead of bilateral surgical exploration (BSE) which is a classical approach in SPA surgery and involves exploration of all parathyroid glands and excision of the pathological one (2).

Measurement of QPTH shows the success of SPA surgical treatment intra-operatively, and is especially performed in the cases who underwent unilateral surgery or minimally invasive method. Intra-operative QPTH measurements in predicting the success of surgery have a sensitivity and specificity of 98% and 94%, respectively, and an overall accuracy of 97% (3). In SPA cases localized by preoperative PS and/or USG, there is a doubt on whether to measure intra-operatively QPTH (4,5,6). The aim of this study was to evaluate the efficiency of parathyroidectomy using GP-MIP without performing intra-operative QPTH measurement in SPA patients in whom the adenoma was localized preoperatively by dual phase 99mTc-MIBI PS and USG. 

## MATERIALS AND METHODS

This clinical study was performed in Okmeydanı Training and Research Hospital between February 2006 and January 2009, with 31 SPA patients (27 female, 4 male; mean age 51±11years, range 31-77 years), whose SPA were localized by dual phase 99mTc-MIBI and USG in preoperative period, and in which GP-MIP was performed without intra-operative serum QPTH measurement. Our patients had primary HPT without persistent-recurrent signs, no history of familial HPT or MEN syndrome, no thyroid nodule in USG and 99mTc-MIBI PS and USG showed a SPA in the same location.

The dual phase 99mTc-MIBI PS was performed as a dual-phase single-tracer examination. Following the intravenous (iv) injection of 740 MBq of 99mTc-MIBI, planar static images of the anterior view of the neck and upper thorax at a matrix size of 128?128 were obtained with the patient in the supine position. Images were obtained at 20 min and at 120 min for the delayed phase. A gamma camera (E-cam, Diacam, Siemens, Chicago, IL, USA) equipped with a low-energy high-resolution parallel hole collimator was used for image acquisition. USG was performed in all patients using 10 MHz transducers (EUB-405 PLUS, Hitachi, Japan). USG was performed and evaluated by the nuclear medicine doctors (S. K., S. S. K.), who also interpreted the dual phase 99mTc-MIBI PS.

Serum PTH levels (normal range 12-88 pg/mL) of the patients were measured by chemiluminescence method in a hormone autoanalyzer using commercial kit (Unicel DXI 800 Synchron system, Beckman Coulter, Fullerton, CA, USA). Serum Ca+2 level was measured by an autoanalyzer using the commercial kit (Roche Diagnostics Corp, Indianapolis, USA), by calorimetric method.

99mTc-MIBI PS and GP-MIP were performed on different days. The GP-MIP started 90-120 min after the iv injection of 740 MBq 99mTc-MIBI as described above. All patients were operated within 30 days after the dual phase 99mTc-MIBI PS examination. Before the incision, counts/ sec were obtained from the four quadrants of the neck every 10 sec. by the gamma probe (GP) device (C-trak system, Care Wise Morgan Hill, California, USA), The incision was performed following local anesthesia, by taking into consideration the maximum upper skin counts and the USG findings. The area where the maximum counts/sec was observed indicated the location of the SPA and where the lesion was excised. The counts, taken from ex vivo SPA (a), and the background counts taken from the area where the lesion was excised (b) were measured. By dividing (a) by (b) and multiplying the quotient by 100%, value of ex vivo SPA counts in every bed were determined. If (a) counts were 20% more than the (b) counts, the excised lesion was accepted as SPA and the operation was terminated.

In all patients, serum PTH and serum Ca+2 levels were measured postoperatively. Patients with no surgical complications were discharged in the postoperative day. Serum PTH and Ca+2 levels were again measured at 1, 6 and 12 months.

The study protocol was approved by our hospital’s Ethics Committee, and all patients provided their written informed consent for performing the study.

## STATISTICS

Data were analyzed using SPSS 15.0 for Windows. Results were expressed as mean ± SD. Comparisons of the data were performed by Mann Whitney-U and Chi-square tests. Correlation analyses of continuous variables were performed by Pearson’s rank test. Results were considered statistically significant when the two-tailed p value was less than 0.05. 

## RESULTS

**Figure 1:**
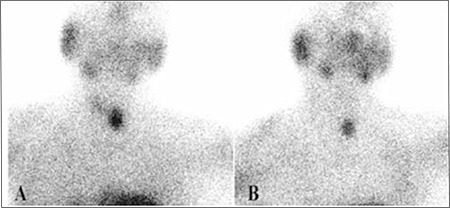
Early (A) and delayed (B) Tc-99m MIBI PS images from a dualphase study show focal uptake and retention of the tracer in the left inferiorbed of the thyroid

In dual phase 99mTc-MIBI PS, maximum neck uptake indicating SPA is observed in the left inferior thyroid bed in 15 patients; in the left superior thyroid bed in 4 patients; in the right inferior thyroid bed in 11 patients, and right superior thyroid bed in 1 patient (Figure 1). The presence of SPA is confirmed by USG.

Intra-operative findings and other features related to the operation in GP-MIP are shown in Table 1. In all cases, except 1 patient, the operation was performed under the local anesthesia due to the enormity of size of SPA, in which general anesthesia is chosen. The GP count value, which was taken from SPA, was found more than 20% of the lesion bed counts in all cases (the lowest rate was 83,5% and the highest 265,9%). The preoperative and postoperative serum PTH and Ca^+2^ levels are shown in Table 2. In all patients, there was more than 50% decrease in PTH levels in the first postoperative day in respect to the preoperative serum PTH levels. When the preoperative and postoperative serum PTH levels were compared by the paired samples T test, there was a statistically significant difference (P=0,001). It was observed that, while the postoperative serum PTH level was normal in 28 patients, it was above the normal level in 3 patients. The postoperative 1st day serum Ca^+2^ levels in all patients has reduced to normal. Thirty patients were discharged from the hospital in the first postoperative day. In one patient, hypocalcemia was detected, then Ca^+2^ replacement was started and she was discharged from the hospital in the postoperative 2nd day. In postoperative 1st month, she was normocalcemic. When the preoperative and postoperative serum Ca^+2^ levels were compared by the paired samples T test, there was a statistically significant difference (P=0,01).

In the early postoperative period, 1 patient experienced seroma in the operation area, which was successfully drained. In the first postoperative month, all patients had normal serum Ca^+2^ and all but one had normal serum PTH levels. In this case; while the patient was also in normocalcemic state at 6th and 12^th^ month controls, serum PTH level was observed to be over the normal limits (preoperative serum PTH: 479 pg/ml, Ca^+2^: 11 mg/dl ; postoperative 12th month serum PTH: 106 pg/ml, Ca^+2^: 9.1 mg/dl). This was accepted as a result of a compensatory response to an abnormality in Ca^+2^ homeostasis because SPA was not detected in USG or 99mTc-MIBI PS.The mean SPA volume was 1882,6±1468,1 mg (range of 400-8000 mg) according to the pathology results. The preoperative serum Ca^+2^ and PTH levels were significantly correlated with the volume of SPA’s (P=0,012 and P=0,02, respectively) as tested by the Spearmen Rank Order Correlations.

## DISCUSSION

Unilateral neck exploration is sufficient for the surgical treatment of the PHPT because the 80-85% of these cases is due to SPA. GP-MIP with or without intra-operative QPTH measurement for PHPT caused by a SPA has been more widely used by the endocrine surgeons, in comparison with BSE (4,5,6,7,8).

QPTH measurement is helpful in confirming the successful removal of the SPA. The MiamiCriterion, i.e., a drop in intact PTH level of 50% or more from the preoperative baseline or the pre-excision level, whichever is higher, 10 min after excision is often used to determine whether a cure has been achieved (9). Although the QPTH measurement is used extensively in the MIP operations, due to the SPA is the factor in most PHPT cases, high success ratios are provided in operations performed without QPTH measurement (10,11,12). The major pitfall of MIP is the risk of missed MGD. The prevalence of multiglandular parathyroid disease among patients with PHPT varies from 1 to 3.5% (13). Thus, when preoperative localization with ^99m^Tc-MIBI PS and ultrasound is concordant for SPA, the use of QPTH is of little value. The harmonization of both the PS and USG examinations in preoperative period for the identification of the SPA may not exclude multiglandular disease (MGD) (14). The benefit of intra-operative QPTH measurement, especially for the detection or excluding of the MGD is emphasized (15,16,17,18,19). The lack of a sufficient decrease in QPTH levels in these cases, suggests that the surgeon should continue exploration (16,17). Despite being very sensitive and easyly performed assay, this method has some drawbacks. In cases of bilateral exploration, patients with MGD have shown an inappropriate 50% drop of PTH values (20,21,22). There is also small but significant false-negative rate and it may lead the surgeon to unnecessary exploration the contralateral side in 5-10% of the cases (23,24). Özimek et al. have found the sensitivity and specificity of %98 and %88 respectively with one false positivity and 6 false negativity of intraoperative QPTH measurement in their study including 235 subjects (25).Agarwal et al’s study yielded that QPTH measurement may not be cost-effective in MIP (26). Failure to detect SPA was between 1-2.7% in patients examined by both 99mTc-MIBI PS and USG, without intra-operative QPTH monitoring (12,27,28). In Kiminori et al’s study, while the cure rate was 93.1% in MIP without intra-operative QPTH measurements; in MIP with QPTH measurement, the cure was 97,5% and revealed that QPTH measurement can elicit especially the PS negative Pas determination (29).

In the retrospective study of Goldstein et al, SPA has been detected by PS in 97 of 100 patients and in these patients, GP-MIP were performed without intra-operative QPTH measurements. The operation has been successful in 94 patients (97%), in 3 patients hypercalcemia continued postoperatively. GP-MIP can be safely performed without intra-operative QPTH measurements in patients who have maximum uptake in PS which is compatible with SPA (8). Jacobson et al have also performed GP-MIP without intra-operative QPTH, 30-60 minutes after the iv injection of 740 MBq of ^99m^Tc-MIBI in 112 patients in whom SPA was detected, and in 110 (98%) of the patients the operation was successful (30). In Suliburk et al’s study with 1020 patients, GP-MIP was performed without intra-operative QPTH measurements with 98% success and they emphasized that the most common cause of failure after MIP is an occult double adenoma (31). Riss et al have reported that the number of patients with persistence after surgery increased significantly from 0.8% to 5.0% without measuring intra-operative QPTH while GP-MIP was only performed in patients in whom SPA was detected by preoperative dual phase 99mTc-MIBI PS and USG. In the above mentioned study, there was no decrease in the intra-operative QPTH level in 15 patients although USG and PS findings were compatible for SPA in the preoperative period; for this reason BSA was chosen in 8 of these patients with a double PA and in 7 patients hyperplastic parathyroid glands were detected (18). Cayo et al have performed parathyroidectomy in a series of 755 patients, intra-operative QPTH monitoring accurately predicted success of the parathyroidectomy in 97.5% (157/161) of patients with MGD. In 37 of patients in this series, upon no decrease of >50% in QPTH level after the single gland excision, surgery was continued and after resection of all hyperfunctioning tissue, there was a decrease of >50% in the QPTH level (19).

In our study, all 31 patients who had concordant findings for SPA by ^99m^Tc-MIBI PS and USG, GP-MIP without measuring the intra-operative QPTH achieved a decrease more than 50% of the PTH levels in the first postoperative day, and no patients relapsed during the one year of follow-up. In our opinion, these results depend mainly on concordant results of the 99mTc-MIBI PS and USG in the preoperative period performed particularly by the same nuclear medicine physicians.

The limitations of this study were the limited number of patients and inclusion of selected patients who have well defined SPA by the 99mTc-MIBI PS and USG. In the future, this method may be applied to larger series with inclusion of parathyroid lesions which are not clearly defined.

In conclusion, according to our results, GP-MIP without intra-operative QPTH measurements might be performed trustfully in PHPT patients who have concordant findings in dual phase 99mTc-MIBI PS and USG technique in locating SPA. 

## Figures and Tables

**Table 1:**
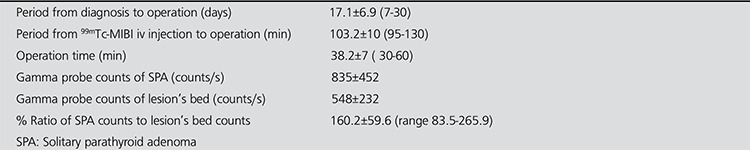
Intra-operative findings and the other features related with the operation

**Table 2:**
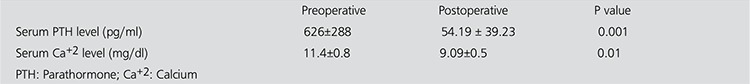
Preoperative and postoperative serum PTH and Ca^+2^
